# A small azhdarchoid pterosaur from the latest Cretaceous, the age of flying giants

**DOI:** 10.1098/rsos.160333

**Published:** 2016-08-31

**Authors:** Elizabeth Martin-Silverstone, Mark P. Witton, Victoria M. Arbour, Philip J. Currie

**Affiliations:** 1 Ocean and Earth Science, National Oceanography Centre, University of Southampton, European Way, Southampton SO14 3ZH, UK; 2 School of Earth Sciences, University of Bristol, Wills Memorial Building, Bristol BS8 1RJ, UK; 3 School of Earth and Environmental Sciences, University of Portsmouth, Burnaby Building, Burnaby Road, Portsmouth PO1 3QL, UK; 4 Paleontology Research Lab, North Carolina Museum of Natural Sciences, Raleigh, NC, USA; 5 Department of Biological Sciences, North Carolina State University, Raleigh, NC, USA; 6 Department of Biological Sciences, University of Alberta, Edmonton, Alberta, Canada

**Keywords:** pterosaur, Azhdarchoidea, Late Cretaceous, Campanian, British Columbia, Northumberland Formation

## Abstract

Pterosaur fossils from the Campanian–Maastrichtian of North America have been reported from the continental interior, but few have been described from the west coast. The first pterosaur from the Campanian Northumberland Formation (Nanaimo Group) of Hornby Island, British Columbia, is represented here by a humerus, dorsal vertebrae (including three fused notarial vertebrae), and other fragments. The elements have features typical of Azhdarchoidea, an identification consistent with dominance of this group in the latest Cretaceous. The new material is significant for its size and ontogenetic stage: the humerus and vertebrae indicate a wingspan of *ca* 1.5 m, but histological sections and bone fusions indicate the individual was approaching maturity at time of death. Pterosaurs of this size are exceedingly rare in Upper Cretaceous strata, a phenomenon commonly attributed to smaller pterosaurs becoming extinct in the Late Cretaceous as part of a reduction in pterosaur diversity and disparity. The absence of small juveniles of large species—which must have existed—in the fossil record is evidence of a preservational bias against small pterosaurs in the Late Cretaceous, and caution should be applied to any interpretation of latest Cretaceous pterosaur diversity and success.

## 1. Introduction

The skies of the Late Cretaceous were home to a clade of extinct, often gigantic flying vertebrates, the azhdarchid pterosaurs. *Arambourgiania philadelphiae*, *Hatzegopteryx thambema* and *Quetzalcoatlus northropi* had wingspans equal to or exceeding 10 m [1–4], and even the smallest known pterosaurs (2.5–3 m, e.g. *Montanazhdarcho minor* [5], *Eurazhdarcho langendorfensis* [6]) from the end of this period had wingspans comparable to the largest extant birds. Smaller pterosaurs are known from the Late Triassic, Jurassic and Early Cretaceous, but it is hypothesized that birds represent the only small-bodied volant vertebrates in the Late Cretaceous [7]. However, a new diminutive pterosaur specimen from the Campanian Northumberland Formation of British Columbia, Canada, with an estimated wingspan under 2 m, demonstrates that this niche was not solely occupied by avians.

**Figure 1.**
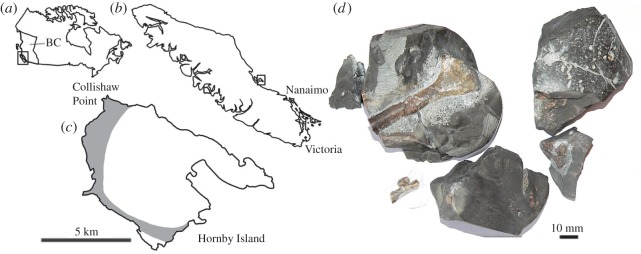
Locality data for RBCM.EH.2009.019.0001, and condition before preparation. (*a*) Location of Vancouver Island in British Columbia, Canada; (*b*) location of Hornby Island off the east coast of Vancouver Island; (*c*) extent of Northumberland Formation and location of Collishaw Point, where RBCM.EH.2009.019.0001A was recovered; (*d*) RBCM.EH.2009.019.0001 as preserved within a carbonate nodule. (*a–c*) after Arbour & Currie [9] and (*d*), photograph by Sandy McLachlan.

Although the west coast of North America is not as rich in Cretaceous terrestrial vertebrates as the Western Interior, multiple fossil-bearing localities from Baja California to Alaska provide terrestrial fossils (see [8] for overview). In particular, the Campanian Northumberland Formation (Nanaimo Group) of British Columbia has produced a diverse array of terrestrial and marine vertebrates. This formation crops out at Collishaw Point, on the northwest edge of Hornby Island, a small island that lies to the east of Vancouver Island in the Strait of Georgia (figure 1*a*–*c*). Fossils from this locality are recovered from carbonate nodules that have weathered out into the intertidal zone. The Northumberland Formation was deposited in a deep-water environment at the edge of a submarine fan [10] and marine fossils such as ammonites, gastropods, crustaceans, teleosts, sharks and mosasaurs are common [11]. Rare terrestrial vertebrate fossils have also been found at this locality, including enantiornithine and ornithurine birds [12,13]. Arbour & Currie [9] identified a pterosaur jaw from Collishaw Point, but Vullo *et al*. [14] have suggested that this specimen is better interpreted as a saurodontid fish, an interpretation supported here. The nearby Cedar District Formation of Denman Island (Upper Campanian, Nanaimo Group) has also produced terrestrial vertebrate fossils, including a non-avian theropod dinosaur vertebra [15], but generally terrestrial vertebrate fossils are rare.

Pterosaur remains are rarely encountered in sediments from the Late Cretaceous west coast of North America. A fourth metacarpal and ulna from two large pterosaurs from the Maastrichtian Chico Formation of California [16] and some fragmentary material from the Upper Campanian El Gallo Formation of Mexico (personal communication in [17]) are the only other known specimens from this time and region. However, pterosaur material is found more commonly (though still infrequently) in the Campanian Dinosaur Park Formation of Alberta [18]. Much of the material that comes out of Alberta is fragmentary and therefore unidentifiable to more detailed taxonomic levels than Pterosauria or Pterodactyloidea *incertae sedis* (E.M.S. personal observation), and those that are identifiable all represent medium to large-size azhdarchids, including *Navajodactylus* [19], and possibly *Quetzalcoatlus* and *Montanazhdarcho* [18]*.* A potential pterosaur manus print is identified from the Upper Campanian/Lower Maastrichtian Wapiti Formation of Alberta [20], while pterosaur tracks have also been reported from the similarly aged Lower Cantwell Formation of Alaska [21,22].

### 1.1. Institutional abbreviations

BSPG: Bayerische Staatssammlung für Paläontologie und Geologie, Munich, Germany; GPIT: Institut für Geologie und Paläontologie, Universität Tübingen, Tübingen, Germany; PMO: University of Oslo Museum of Natural History, Oslo, Norway; RBCM: Royal British Columbia Museum, Victoria, British Columbia, Canada; SMNS: Staatliches Museum für Naturkunde, Stuttgart, Germany; TMP: Royal Tyrrell Museum of Palaeontology, Drumheller, Alberta Canada.

## 2. Material and methods

RBCM.EH.2009.019.0001 consists of 10 pieces (elements A–J) that were found within a single small nodule (8–10 cm across) at Collishaw Point in 2008 by Sandy McLachlan (figure 1*d*). Only elements A–H were studied. The specimens are preserved three-dimensionally, and some have been left partially embedded in the surrounding matrix following their mechanical preparation. All elements are worn, broken and incomplete, making it difficult to ascertain the extent of some bones against surrounding matrix. Although a few elements split when the nodule encasing the fossils was opened, the preservation of the bone surfaces and edges indicate that most damage occurred prior to fossilization. Repair and protection of RBCM.EH.2009.019.0001 has been carried out using resin. In some places, this obscures minor details, textures and morphology.

X-ray computed tomography (CT) scans of the specimens were attempted using a SkyScan 1174 micro-CT scanner at the University of Alberta. The scans were viewed using Mimics x64 14.01 but proved uninformative because of low contrast within the specimens, and are not discussed further here. A transverse thin section was made of the humeral diaphysis of RBCM.EH.2009.019.0001A to study the bone histology. This thin section was made following standard techniques, using a circular rotating disc to cut the diaphysis. The small section was then embedded in resin and processed for thin sectioning to 80 µm thick, using the method described by Chinsamy & Raath [23]. The thin section was then studied under crossed plane polarized light and crossed polarized light.

## 3. Systematic palaeontology

Pterosauria Kaup, 1834 [24]

Pterodactyloidea Plieninger, 1901 [25]

Azhdarchoidea Nessov, 1984 [26] (*sensu* Unwin [27])

Neoazhdarchia Unwin, 2003 [28]

?Azhdarchidae Nessov, 1984 [26]

### 3.1. Description

**Figure 2.**
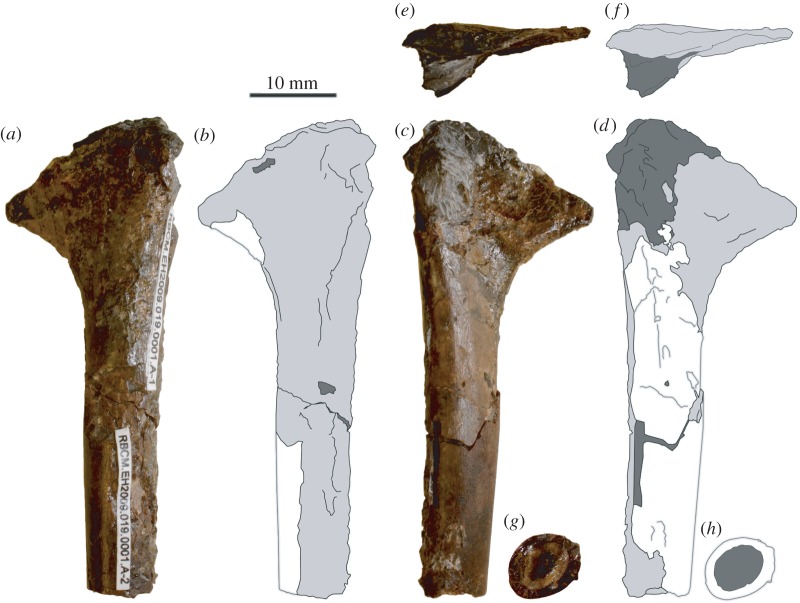
Photographs and interpretative drawings of RBCM.EH.2009.019.0001, element A, a left humerus, in (*a*,*b*) dorsal, (*c*,*d*) ventral, (*e*,*f*) proximal and (*g*,*h*) distal aspect. Shading denotes preserved bone cortex (white); weathered bone (light grey) and matrix (dark grey).

RBCM.EH.2009.019.0001A (element A) is a left humerus and is the best preserved and most diagnostic element found in the nodule (figure 2). The humerus is missing both extremities and has a preserved length of 54.2 mm. Uniquely for this specimen, diagenetic calcitic cements have precipitated in the diaphysis. Based on comparisons with other more complete azhdarchids, the humerus had a total length of about 75 mm. Most features of the humerus are damaged: the ulnar crest and humeral head are absent, the deltopectoral crest is broken along the proximal and anterior borders, and compacta is missing in several regions. The ventral surface has suffered especially in this regard, the deltopectoral crest being reduced to about 1 mm depth in regions where the bone wall is missing. The deltopectoral crest projects anteriorly from the dorsal region of the diaphysis, and does not curve or warp around the shaft of the humerus. In ventral view, the deltopectoral crest is triangular as preserved but the proximal and anterior edges are broken and the actual shape cannot be ascertained. This crest occupies less than one-third of the preserved humeral length (about 18 mm). The diaphysis is parallel sided for its entire preserved length, but deepens in the region distal to the missing ulnar crest. Muscle scars common to the distal diaphysis of three-dimensionally preserved pterodactyloid humeri (e.g. [29,30]) are not discernable. No pneumatic foramina are preserved, but areas where these are typically located in pterosaur humeri are missing or damaged. In cross section, the diaphysis is oval with a minimum width of 6.22 mm. The cortical thickness of the bone wall ranges from 0.82 to 1.25 mm. The external bone surface is better preserved on the shaft than elsewhere on the specimen, and is smooth, but with a fibrous texture typical of immature pterosaur bone [31].

**Figure 3.**
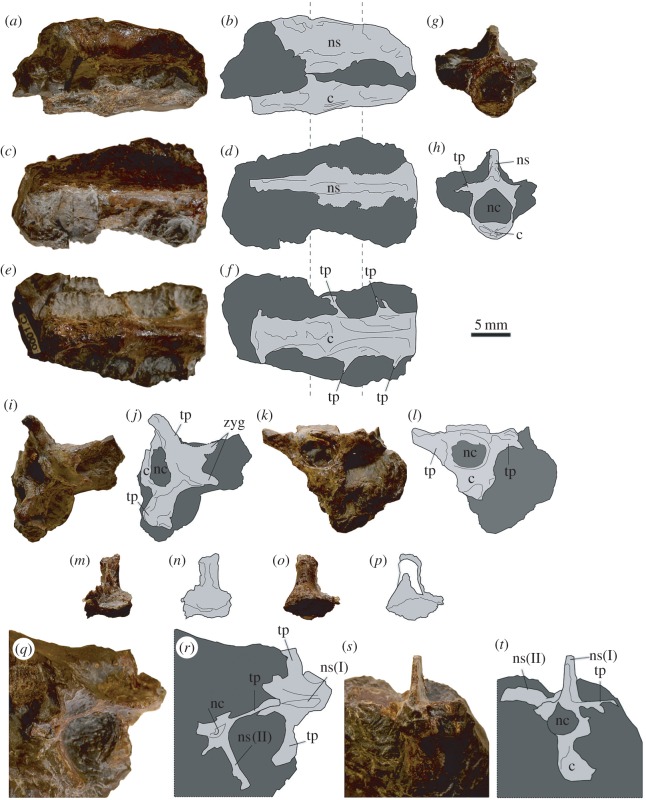
Photographs and interpretative drawings of RBCM.EH.2009.019.0001 vertebral material. (*a–h*) Element C, fragment of notarium in (*a,b*), lateral; (*c,d*), dorsal; (*e,f*), ventral and (*g,h*), anterior aspect; (*i–l*) element D, partial dorsal vertebra in (*i*,*j*), dorsal and (*k*,*l*) posterior aspect; (*m–p*), element G, probable vertebral process, posterior(?) and (*o,p*), anterior(?) aspect; and (*q–t*) element E, two associated dorsal vertebrae in (*q,r*), dorsal and (*s,t*), anterior aspect (c, centrum; nc, neural canal; ns, neural spine; tp, transverse process). Approximate junctions between vertebrae of element C are indicated by dotted lines.

Three fused vertebrae are present in RBCM.EH.2009.019.0001C (element C, figure 3*a*–*h*), representing part of the notarium, a fused series of dorsal vertebrae in the pectoral region of some pterosaurs. These vertebrae can be distinguished from fused sacral vertebrae because the large intervertebral openings common to pterosaur sacra are not discernable despite the large height of the preserved neural spines [29,32–34]. Intervertebral openings can be considerably reduced in pterosaur notaria, sometimes present only below the neural arch [29,35,36], and this morphology is consistent with element C. These vertebrae are extensively weathered, missing the distal portions of the neural and transverse spines, and in some areas, the depth of the centra has been reduced considerably. Extrapolating the depth of the posterior two centra to the most anterior suggests at least 2 mm have been worn off the ventral surface of this vertebra, almost half the estimated height of the original centrum. The broken dorsal margins of the neural spines are rounded and partially obscured by resin. As per other pterosaur notaria [35,36], posterolateral projection of the transverse processes allows for this element to be oriented, the tallest (as preserved) vertebra corresponding with the posterior end. The preserved series is 21 mm long, with each vertebra measuring approximately 7 mm in length—the exact margins are difficult to discern given the entirely ankylosed and weathered nature of the specimen. The entirety of the centra and neural spines are co-ossified. The centra are gracile, being approximately twice as long as wide, with constricted mid-lengths. The posteriormost centrum has prominent oval sulci on the lateral surfaces, and all three possess a continuous, slightly prominent ridge along the ventral margin. No prezygapophyses or postzygapophyses are visible. In anterior aspect, the transverse processes project perpendicular to the neural spines. They are largely obscured by matrix in dorsal or ventral aspects, but can be seen as extending posterolaterally, oriented and being anteroposteriorly broad dorsally, and tapered ventrally along their lengths.

RBCM.EH.2009.019.0001D (element D, figure 3*i*–*l*) is a partial dorsal vertebra. The centrum is slightly oval in shape and has a large, oval sulcus on the left side. The condyle is 4 mm high, 4 mm wide, and has a square profile. Both transverse processes are preserved and are approximately 4.5 mm long and strongly deflected posteriorly. The right transverse process has a broadly expanded tip. Although the neural spine is missing, the neural canal is visible in posterior aspect, and is a large, oval aperture 4.7 mm wide and 3.7 mm high. The neural spine of element D is potentially represented by RBCM.EH.2009.019.0001G (element G, figure 3*m*–*p*). This element is a small, disassociated vertebral process with a square base that has a robust spine of subquadrangular aspect. The tip is missing, and resin adheres to the (assumed) posterior and proximal faces. The broken margin of element G roughly matches the broken dorsal surface of the element D vertebra.

RBCM.EH.2009.019.0001E (element E, figure 3*q*–*t*) includes two partially exposed dorsal vertebrae. They are obliquely arranged with respect to each other, the neural arch of one abutting the transverse process of the other. The anterior and dorsal regions of one vertebra are exposed, with all surfaces being weathered or broken. The right transverse process of this vertebra is almost complete and expanded distally and posteriorly, and the left transverse process is similar except for the missing distal end. The centrum is tall and narrow, with a poorly preserved portion of the neural canal suggesting a rounded cross section. The neural spine is tall but the exact shape is not discernible because it is obscured by matrix. The second vertebra is only partially discernible in lateral and posterior aspect. The neural spine is large, apparently nearly complete, and has a square profile in lateral view. The left transverse process projects posterolaterally, expanding somewhat towards the distal end. The posterior opening of the neural canal is rounded with an exposed opening about 1 mm in diameter.

**Figure 4.**
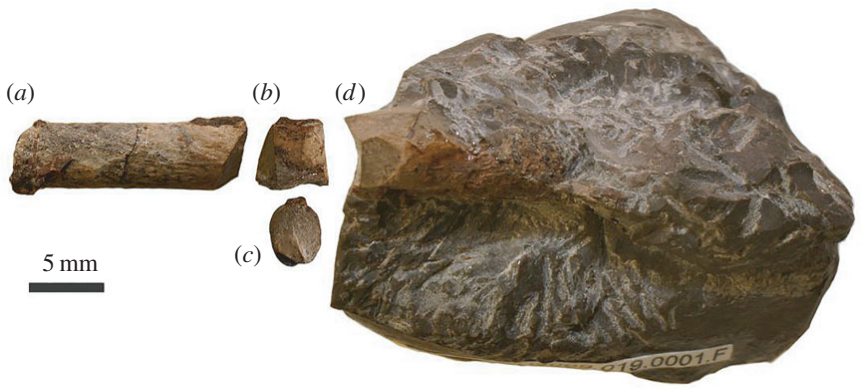
Photographs of unidentified material of RBCM.EH.2009.019.0001, mostly comprising internal moulds of a long bone. (*a*) element B; (*b,c*) element H in lateral(?) and anterior(?) aspect; (*d*) element F.

The identities of RBCM.EH.2009.019.0001B, F and H (figure 4) cannot be determined. Each comprises a partial, tubular internal mould of a long bone of oval cross section and similar diameter (approx. 5 mm). The colour, shape and lithology of these elements match and probably represent remains of one bone, perhaps pertaining to a forearm element.

### 3.2. Bone histology

To assess the ontogenetic stage of the Hornby specimen, we created a thin section of the RBCM.EH.2009.019.0001A humeral shaft close to its broken distal margin. This represents the best and most completely preserved region of bone on the humerus and, with the shaft being unexpanded, we assume the section records the relatively ontogenetically stable diaphyseal region rather than the continually reworked metaphysis.

**Figure 5.**
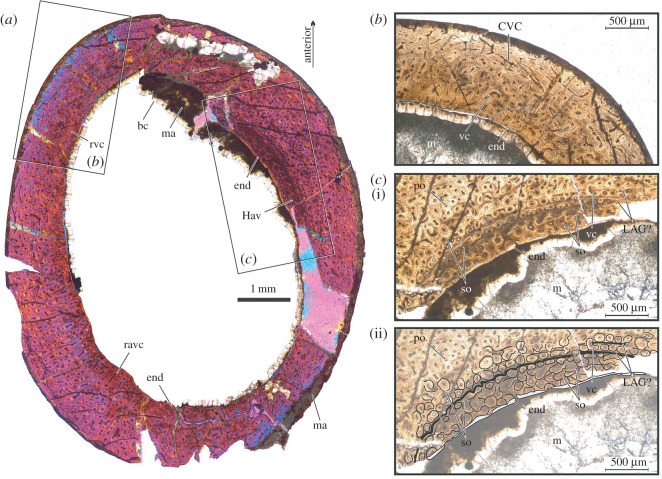
Histology of RBCM.EH.2009.019.0001A, left humerus, transverse cut through distal diaphysis. (*a*) Entire thin section in cross polarized light; (*b*) detail of cortical bone showing differentiation of highly vascularized reticular inner regions, and relatively avascular outer layer with laminar vascular canals, normal light, (*c*) detail of thickened and reworked endosteal region, thought to represent the base of a trabeculum or pneumatic structure, and possible evidence of zonal growth ((i) photograph in normal light; (ii) as (i) but superimposed with interpretative line drawing of major features). For clarity, only select features are labelled (bc, bladed calcite cement; CVC, circumferential vascular canals; end, endosteal lamella; Hav, Haversian system; LAG, line of arrested growth; m, medullary cavity; ma, sediment matrix; po, primary osteon; ravc, region of radial vascular canals; rvc; reticular vascular canals; so, secondary osteon; vc, vascular canal).

The thin section shows that the humerus is composed of vascularized fibrolamellar bone with localized reduction of vascularization in some parts of the outer cortex and many well-defined primary osteons visible throughout (figure 5*a*). The external bone surface shows evidence of some bacterial invasion, similar to that seen in *Tenontosaurus* [37], but nevertheless reveals that few vascular canals access the periosteal surface (figure 5*b*). The majority of canals and osteocyte lacunae in the outer cortex are oriented circumferentially, whereas canals located elsewhere in the cortex are mostly arranged in a reticular pattern. These deeper cortex canals show greater width than those adjacent to the periosteal surface. A thin endosteal lamella can be seen wherever the inner regions of cortical bone are undamaged, and contains numerous osteocyte lacunae. The endosteal lamella cuts through a number of osteons, recording erosive expansion of the medullary cavity.

The cortex is locally expanded by the addition of a 325 µm thick lens of densely packed primary and secondary osteons in the anteroventral endosteal region (figure 5*c*). A number of overlapping osteons can be observed in this region (figure 5*c*ii) that seem to be localized development of dense Haversian tissue similar to that reported in endosteal bone for other pterosaurs [38]. Secondary osteons are primarily concentrated at one end of the thickened region, but also occur along the boundary between reworked and cortical bone. Concentrations of Haversian bone are known to occur in endosteal pterosaur bone where trabeculae or pneumatic structures attach to cortical bone [38]: this explains the localized endosteal thickening in RBCM.EH.2009.019.0001. The locally expanded cortex could be attributed to the non-midshaft location of thin section, if our section represents a growth zone [39].

Two (approx. 20 µm) circumferential layers of bone occur within the cortex adjacent to the region of Haversian bone (figure 5*c*). The innermost of these is more substantially represented and is largely laminar in structure, but undulates around the margins of primary osteons in places. The outer layer has been substantially reworked to the extent that it is almost entirely obliterated, but is laminar for its visible length. It does not undulate around primary osteons, but also does not cut across any. These details match descriptions of lines of arrested growth (LAGs) seen in other pterosaur bones [38,40], and these structures probably represent the results of zonal growth, perhaps LAGs.

## 4. Discussion

### 4.1. Taxonomic affinities

The bones comprising RBCM.EH.2009.019.0001 were found associated with each other in a single nodule, but because they are disarticulated it is possible that they represent multiple individuals or multiple taxa. However, commonality of preservation, the lack of overlapping elements, identification of bones closely associated in an articulated skeleton (a humerus, notarial vertebrae and dorsal vertebrae) and the small size of the nodule suggests a single source for the remains. Consistent indications of small size and similar phylogenetic characteristics across the new material also favour the conclusion that all of RBCM.EH.2009.019.0001 represents one individual.

The thin bone walls, gracile bone construction and humeral morphology of RBCM.EH.2009.019.0001 indicate it clearly belonged to a volant Mesozoic animal, a pterosaur or avialan. Several features of RBCM.EH.2009.019.0001 indicate a pterosaurian identity. In particular, the proportionally large, tongue-like deltopectoral crest, which strongly projects anteriorly from a straight, slender humeral diaphysis (element A), is a pterosaurian trait. The dorsal vertebrae possess pterosaurian features including proportionally thin bone cortices, gracile bone construction, excavated corporeal cavities and fusion of several elements [28,39]. Additionally, several features argue against an avialan identity. The deltopectoral crests of most Mesozoic birds are, like modern forms, generally less prominent than those of pterosaurs and typically dorsally deflected. This produces a ‘sigmoidal’ profile in proximal view (e.g. [41]) which contrasts with the proximal aspect of pterosaur humeri (figure 2*e,f*). Some avialans, such as *Confuciusornis* and *Ichthyornis* have prominent deltopectoral crests [41], but their proximal humeral morphology also strongly differs from those of pterosaurs and this new specimen. The dorsal vertebrae of RBCM.EH.2009.019.0001 are longer and taller than those of many Mesozoic birds (see examples in [42]), but consistent with those of pterosaurs [29,42]. Finally, the presence of a notarium is more representative of a pterosaur than a Late Cretaceous bird [41]. A pterosaur identity is most likely for RBCM.EH.2009.019.0001.

The proportionally thin bone cortices of the vertebrae and humerus are typical of pterodactyloid pterosaurs [28,39], although they alone do not rule out a non-pterodactyloid identity [43]. However, given that no non-pterodactyloid clades have been positively identified from the Cretaceous, it is most probable that RBCM.EH.2009.019.0001 represents a pterodactyloid pterosaur.

The humerus (element A) provides the most information regarding the pterodactyloid affinities of RBCM.EH.2009.019.0001. The deltopectoral crest is unlike the long, warped deltopectoral crest seen in most ornithocheiroids *sensu* Unwin [28], and bears no suggestion of the ‘hatchet-shape’ typical of deltopectoral crests in nyctosaurid ornithocheiroids [17,28]. Element A also lacks a continuously distally expanding diaphysis typical of ornithocheiroids [30]. The humeral shaft of non-pterodactyloid pterosaurs and ctenochasmatoids is often bowed [28,39,44]. The shaft of element A, as preserved, is straight, similar to humeri referred to Azhdarchoidea and Dsungaripteroidea (both *sensu* Unwin [27]) and the controversial pterodactyloid genus *Lonchodectes* [28,39]. However, the broken proximal head is not representative of *Lonchodectes,* and some studies doubt the existence of the Lonchodectidae and validity of *Lonchodectes,* suggesting humeri referred to this genus actually pertain to indeterminate azhdarchoids [45,46]. The cortical thickness of the shaft is approximately 1 mm, and thus thinner than the secondarily thickened compacta of Dsungaripteroidea [28].

An azhdarchoid identification is most likely for element A, with the unwarped deltopectoral crest, parallel-sided and straight shaft, and slender bone walls being typical of humeri in these large-headed, toothless pterosaurs [28,30,44,47]. The lack of diaphyseal expansion in element A corresponds particularly well with the humeri of several neoazhdarchian azhdarchoids (Thalassodromidae + Azhdarchidae, *sensu* Unwin [28]) such as the ‘Wessex’ humerus [30], the ‘Glen Rose’ humerus [48]; *Azhdarcho lancicollis* [49] and *Quetzalcoatlus northropi* [50]. It is of interest that the oval cross section of the humeral diaphysis is not oriented, as in most pterosaurs, with the long axis perpendicular to the deltopectoral crest. A similar morphology occurs in the holotype humerus of the giant azhdarchid *Hatzegopteryx thambema* [3]. This was considered a consequence of distortion by Witton & Habib [4], but the observation of a similar arrangement in RBCM.EH.2009.019.0001 might suggest this is a genuine feature of some neoazhdarchian humeri. If so, it provides another reason to assume RBCM.EH.2009.019.0001 has a neoazhdarchian affinity. One potential issue with this identification is that the relative cortical thickness of the shaft of the humerus is thicker in relation to the diaphysis than most azhdarchoid specimens. We calculate the air space proportion (ASP, the relative amount of space in a bone occupied by air [51]) in the humerus of RBCM.EH.2009.019.0001 as 0.46, lower than in other azhdarchoids and pterodactyloid humeri and wing bones ([52], table 1). However, somewhat thickened bone walls are known from some azhdarchoids [3] and, moreover, measurements of cortical thicknesses across pterosaurs suggest smaller individuals have lower ASP than larger ones (table 1). The effect of body size on ASP is currently being explored further by E.M.S. and others and is provisionally thought to reflect biomechanical constraints (e.g. a minimal cortical thickness required to maintain functionality in small pterosaur bones [54]) rather than having taxonomic significance.

**Table 1.** ASP values in various pterosaur elements. Azhdarchoidea and Ornithocheiroidea represent large-bodied taxa (wingspan >4 m) while the remainder are classified as ‘small-bodied’.

group	source	humerus	radius	ulna	wing phalanges
Hornby humerus	RBCM.EM.010.0001A	0.46			
**Azhdarchoidea**					
*Hatzegopteryx*	Witton & Naish [53]	0.82^a^			
*Quetzalcoatlus*?	TMP 1992.83.4	0.85			
*Bennettazhia*	Martin & Palmer [52]	0.81			
**Ornithocheiroidea**					
*Pteranodon*	Martin & Palmer [52]	0.90	0.81	0.90	0.74–0.88
Ornithocheiridae indet.	Martin & Palmer [52]				0.68–0.83
Pterodactyloidea indet.	SMNS 81976	0.86^b^			
**small-bodied**					
*Dorygnathus*	GPIT RE/08048				0.46
*Rhamphorhynchus*	BSPG 1938 I 503				0.34^b^
*Germanodactylus*	BSPG 1892 IV 1/BSPG 1977 XIX 1		0.49^b^	0.35^b^	0.36–0.54^b^
unknown	PMO 162.882				0.52–0.61

^a^Specimens with ASP calculated from published cross sections.

^b^ASP values estimated from *K*^2^, where *K* is the ratio of inner to outer diameter of a bone.

Other elements of RBCM.EH.2009.019.0001 are of less systematic utility, either being too poorly preserved (elements B, F and H) or from skeletal components with limited diagnostic potential (elements C, D and E). However, the fusion of three vertebrae in element C is consistent with the neoazhdarchian identity suggested, one characteristic of these pterosaurs being the development of a notarium [28]. Ankylosed dorsal vertebrae are not unique among pterodactyloids to neoazhdarchians, but is consistent with the humeral morphology also preserved in RBCM.EH.2009.019.0001 and thus indirectly supportive of this identification.

A neoazhdarchian affinity for the Hornby Island material matches current understanding of Campanian–Maastrichtian pterosaur diversity. One group of azhdarchoids—the neoazhdarchian clade Azhdarchidae—dominates this interval of pterosaur evolution. Campanian–Maastrichtian azhdarchids have a nearly cosmopolitan fossil record and at least 10 species are known from localities in Asia, Europe, North America and South America (e.g. [55]). Non-azhdarchids from the Late Cretaceous are comparatively rare (e.g. [17,56,57]) and none are confirmed from the Campanian or Maastrichtian (approx. 80–66 Ma) of North America. Indeed, only a possible nyctosaurid humerus from the Maastrichtian of Brazil [58] and an alleged fragmentary ornithocheiroid from the Campanian of Montana [59] indicate the presence of non-azhdarchid pterosaurs in this interval. The only pterosaur remains positively identified thus far from North America at this time are azhdarchids [5,18,19,60]. Given this record and the neoazhdarchian features of RBCM.EH.2009.019.0001, the Hornby pterosaur probably represents an additional Campanian azhdarchid, although more substantial and characteristic remains are needed from the Northumberland Formation to confirm the presence of the group in this region.

### 4.2. Ontogenetic status

RBCM.EH.2009.019.0001A is one of the smallest Late Cretaceous pterosaur humeri currently known, and determining the ontogenetic stage of this individual is important for our understanding of body size diversity in Late Cretaceous pterosaurs. Pterodactyloid growth regimes have been well studied in recent years to the extent that the ontogenies of specific clades—including azhdarchids—are increasingly well known [37,38,61,62]. This permits ontogenetic insights into even fragmentary specimens like the new Hornby material.

Gross surface morphology and microstructure of the humerus indicate this individual was still growing at time of death. Externally, the diaphysis of the humerus has a striated, fibrous texture characteristic of immature pterosaurs [31], dinosaurs [63] and birds [64], and this texture is reflected internally by the largely reticular fibrolamellar bone texture. These features only provide coarse insight into ontogeny, however, as such bone is retained until relatively late/‘subadult’ ontogenetic stages in most pterodactyloids, including azhdarchids [31,62]. Other details of the humeral microstructure indicate with more specificity that the animal was not a young juvenile. The periosteal region is not accessed by widely open vascular canals as seen in neonate or perinate pterosaurs (see fig. 1 of [40] for comparison), and a transition from a reticular fibrolamellar bone to laminar periosteal bone—this being a feature of mature pterosaurs—is underway in several regions [40,62]. The evidence of resorbed endosteal surfaces and possible signs of zonal bone growth are further signs that RBCM.EH.2009.019.0001 was not an especially young animal. Secondary remodelling related to large endosteal structures and the presence of numerous secondary osteons are features only occurring in late-stage juvenile or subadult pterodactyloids [38].

The endosteal lamella provides particular insight into ontogenetic stage. It is being recognized that pterosaur growth strategies may have been varied and that the significance of endosteal structures may differ for certain clades [62]. For at least *Pterodaustro*, *Pteranodon* and azhdarchids, endosteal lamellae correlate with cessation of medullary expansion [61,62]. If our identification of RBCM.EH.2009.019.0001 as an azhdarchoid is correct, the presence of an endosteal lamella probably indicates the medullary cavity had stopped growing. Furthermore, endosteal lamellae are widely reported as mature features of pterodactyloid bone [38,40,63,64]. They are only attained by *Pterodaustro* individuals over 53% of adult size [61], and we stress that this pterosaur seems to acquire endosteal bone relatively early in ontogeny: they do not occur in other pterodactyloids (including azhdarchids) until much later, even subadult stages of growth [38,62].

We interpret these histological features as indicating RBCM.EH.2009.019.0001 was a late-stage juvenile or subadult [65]. The fact that pterosaur humeri were continually and extensively remodelled during growth makes them suboptimal bones for determining ontogenetic stage (their histology can be complex and difficult to interpret thanks to features like locally expanded cortices, an absence of reversal lines, etc.), but the presence of an endosteal lamella and secondary osteons indicate that this specimen was probably not a young juvenile. These features have been identified as representing later-stage juveniles or subadults in a variety of bones from other pterodactyloids (including forelimb material) [31,38,61,62] as well as comparably aged birds (*Hesperornis* [66]) and non-avian dinosaurs (*Tenontosaurus* [37]).

Corroboration of this ontogenetic stage is seen in other aspects of the Hornby specimen. All RBCM.EH.2009.019.0001 vertebrae have fused neural arches and centra, and at least three are ankylosed into notarial vertebrae. The significance of notarial fusion in ontogeny remains to be fully understood, but notaria are generally thought to have formed during later growth stages. Bennett [31] suggested that notarial fusion (along with several other postcranial fusions) might start during earlier stages of osteological maturation, while Kellner [67] noted that notarial formation can continue beyond development of an otherwise entirely fully mature skeleton. Given the variation seen in other aspects of pterosaur growth, it is possible this feature developed variably in different pterosaur species. Nevertheless, pterodactyloids are not known to fuse notarial elements early in development, and some delayed notarial fusion even once large size (wingspans exceeding 4 m) had been reached [42,68].

Pterosaur specimens, like those of other ornithodirans, can show different ontogenetic signals in different parts of the skeleton (e.g. element fusion, bone texture, size [69,70]), so the vertebral characteristics of RBCM.EH.2009.019.0001 are useful corroboration of histological evidence about the growth stage of this individual. We conclude that the small size of RBCM.EH.2009.019.0001 is only partly related to its immaturity: numerous anatomical and microstructural hallmarks suggest that this animal was a late-stage juvenile or subadult approaching osteological maturity and was unlikely to ever be a large animal, even at full size.

### 4.3. Implications for Late Cretaceous pterosaur body size diversity

Pterosaurs from the latest Cretaceous were typically large, and small pterosaurs from this time period are exceedingly rare. Medium-sized pterosaur species with wingspans of 2.5–3 m are common in Late Cretaceous pterosaur faunas, representing approximately 70% of all finds from pterosaur-productive Maastrichtian localities in Romania [71,72]. Also present in Campanian–Maastrichtian terrestrial ecosystems were truly gigantic pterosaurs with wingspans up to 10 m [1–4]. Conspicuously absent in Late Cretaceous deposits—and, indeed, rare throughout the Cretaceous generally—are fossils of small (less than 2 m) pterosaurs and early stage juveniles of large-bodied taxa [7,73]. In order to compare the body size of RBCM.EH.2009.019.0001 with other pterosaurs, a regression equation of humerus to wingspan measurements was calculated using a dataset of 11 complete azhdarchoid wing skeletons (table 2). ‘Wingspan’ is treated here as the combined length of all forelimb wing elements (minus carpals) multiplied by two per [29]. The equation (*R*^2^ = 0.9868) used is

**Figure 6.**
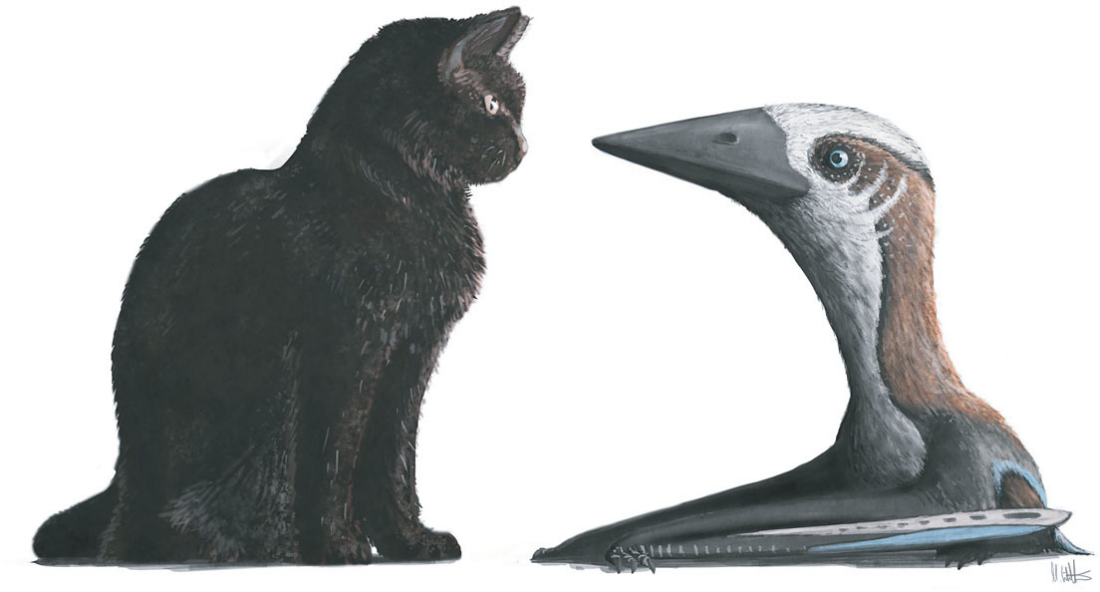
Speculative restoration of a 1.4 m wingspan azhdarchid, representing the atypically diminutive Late Cretaceous azhdarchoid specimen RBCM.EH.2009.019.0001, against a modern housecat (*ca* 300 mm tall at shoulder). All other Campanian and Maastrichtian azhdarchids are famous for being much larger, the biggest being as tall as giraffes and, even at their smallest, comparable in size to the largest extant flying birds. The pterosaur is restored here with anatomical characteristics and body proportions predicted for neoazhdarchian and azhdarchid azhdarchoid pterosaurs [30,42].

4.1 $$\text{Wingspan}=22.535h^{0.9534},$$

where *h* is humeral length (mm). From this, we predict RBCM.EH.2009.019.0001 had a minimum wingspan of 1.03 m based on the preserved humerus length, and 1.4 m for a reconstructed 75 mm length. A similar wingspan estimate is suggested by the RBCM.EH.2009.019.0001 vertebrae. At around 7 mm long each, the vertebrae of this specimen are comparably sized to one of the smallest Lower Cretaceous azhdarchoids known, *Vectidraco*, an animal cautiously estimated to span 0.75 m across the wings [34]. They are also comparable in size to the vertebrae of the Jurassic pterodactyloids *Cycnorhamphus suevicus* and *Herbstosaurus pigmaeus*, which have estimated wingspans of 1.6 m and 1.5 m, respectively [83,84]. Given that several aspects of RBCM.EH.2009.019.0001 anatomy and microanatomy seem to indicate an advanced stage of growth, it is probable this individual would not have grown significantly beyond these estimated dimensions (figure 6).

**Table 2.** Azhdarchoid forelimb bone lengths used in wingspan regression of RBCM.EH.2009.019.0001.

					wing phalanges (mm)				
taxon (specimen number)	references	humerus (mm)	ulna (mm)	Metacarpal IV (mm)	1	2	3	4	estimated wingspan (m)
*Sinopterus jii* (GMN-03-11-001)	[74]	79	117	132	163	127	91	41	1.50
‘*Huaxiapterus*’ *corollatus* (ZMNH M8131)	[75]	80	114	152	176	109	69	34	1.47
*Sinopterus dongi* (IVPP V13363)	[76]	59	88	95	121	88	63	32	1.09
*Sinopterus dongi* (D2525)	[77]	108	154	169	215	156	106	43	1.90
*Eoazhdarcho liaoxiensis* (GMN-03-11-002)	[78]	90	122	135	178	139	93	50	1.61
*Eopteranodon lii* (D2526)	[79]	68	93	105	137	103	75	62	1.29
*Jidapterus edentus* (CAD-01)	[80]	80	112	147	177	120	73	40	1.50
*Shenzhoupterus chaoyangensis* (HGM 41HIII-305A)	[81]	66	105	140	147	100	68	36	1.32
*Quetzalcoatlus* sp. (TMM 42422)	[82]	250	358	620	602	305	156	39	4.66
*Tupuxuara leonardii* (IMCF 1052)	[82]	234	291	359	505	301	208	40	3.88
*Microtuban altivolans* (SMNK PAL 6595)	[56]	73	92	122	135	115	64	4	1.21

RBCM.EH.2009.019.0001 is therefore one of the smallest Campanian–Maastrichtian pterosaur specimens known, perhaps comparable in size to just three contemporary specimens: two tiny azhdarchid cervical vertebrae from Dinosaur Park Formation, Alberta (the most complete of which is just 88 mm long; [18,102]) and *Piksi barbarulna* from the Campanian Two Medicine Formation of Montana, known from small, fragmentary forelimb elements and originally identified as an ornithocheiroid with a 1 m wingspan [59]. Whether *Piksi* actually represents a small pterosaur is debatable, as its pterosaurian characterization is problematic. Several details of the distal humerus differ from all known pterodactyloid humeri, including the proportional dominance of the entepicondyle; the near-perpendicular orientation of the capitulum; the deep olecranon fossa; a strongly convex distal margin; swollen posterior tuberosity; marked asymmetry in distal view; and the obtuse angle between the distal margin versus the dorsal margin. These features occur in some theropods [85], but are atypical of all pterodactyloid humeri. They perhaps indicate that *Piksi* is not a diminutive, late-surviving ornithocheiroid pterosaur as recently suggested [59].

The scarcity of small-bodied pterosaur species has been interpreted as the absence of these animals altogether in Campanian–Maastrichtian pterosaur faunas, despite the abundance of pterosaur species with wingspans under 2 m in the Jurassic (e.g. [7,39,73,86]). RBCM.EH.2009.019.0001 highlights the perplexing circumstances surrounding the absence of small pterosaurs at the end of the Mesozoic. This is often interpreted as a component of gradual pterosaurian decline towards the end of the Cretaceous, the loss of small species coinciding with reduced taxic diversity and morphological disparity [7,39,87–91]. Some controversy exists over the cause of this apparent decline. Benson *et al*. [7] suggest that direct competition from birds displaced smaller pterosaur species by the end of the Mesozoic, while McGowan & Dyke [92] suggest that birds and pterosaurs did not occupy the same niches. Other studies have not found that pterosaur diversity declined inversely with neornithine bird diversification [87,93].

A key, seemingly overlooked factor in any interpretation of this phenomenon concerns the absence of hatchling or even small juvenile pterosaur remains from the latest Cretaceous. These small-bodied individuals must have existed, and yet seldom occur as fossils. There are some records of immature giant pterosaurs, such as certain *Arambourgiania* remains from the Maastrichtian of Jordan [94], and the possible small azhdarchid cervical vertebra from Alberta [18,102], but these are extremely rare compared with the remains of larger individuals. Their absence is almost certainly a preservational bias against small animals, including pterosaurs, in the latest Cretaceous, be they juvenile individuals or small adults. This proposal is not without precedent: for example, the Dinosaur Park Formation of Alberta exhibits a strong preservational bias against small-bodied and embryonic or juvenile dinosaur taxa [95]. Further evidence for a large preservational bias in the pterosaur fossil record is evident from Lagerstätten effects which overwhelm our understanding of pterosaur evolution and undermine our perception of intervals without exceptional preservation—such as the Late Cretaceous [90]. Well-documented shifts in preferred pterosaur habitats and ecologies may partly explain these biases: less than half of Cretaceous pterosaurs occurred in marine environments, versus more than 70% in the Triassic and Jurassic [96], and Late Cretaceous taxa seem particularly well adapted to life in terrestrial settings [53,97,98]. Indeed, several studies have noted that azhdarchids, the dominant pterosaur clade of the Late Cretaceous, occur primarily in non-marine settings [53,99,100]. If Cretaceous pterosaurs had largely moved to more terrestrial environments where preservation is less common, both their apparent decrease in diversity and reduced occurrences of small forms might reflect preservational or taphonomic biases against smaller, less readily preserved pterosaur skeletons. The diminutive pterosaur fossils from the Campanian Northumberland Formation of British Columbia might suggest small pterosaurs were present in some capacity at the end of the Cretaceous but, as fossils at least, they remain extremely rare.

RBCM.EH.2009.019.0001 represents the first record of azhdarchoid pterosaurs from British Columbia and adds to the relatively sparse record of Campanian pterosaurs in Canada. Representing one of the smallest known pterosaurs from Campanian–Maastrichtian strata, it adds to a growing set of evidence that latest Cretaceous pterosaur faunas were not dominated by large or giant species, and that smaller pterosaurs may have been well represented in this interim (e.g. [72,101]). As with other evidence of smaller pterosaurs from the latest Cretaceous, RBCM.EH.2009.019.0001 is fragmentary and poorly preserved: researchers should check collections more carefully for misidentified or ignored pterosaur material which may enhance our picture of pterosaur diversity and disparity at this time.

## Data accessibility

There are no additional data for this study.
